# Pathological Lying: Theoretical and Empirical Support for a Diagnostic Entity

**DOI:** 10.1176/appi.prcp.20190046

**Published:** 2020-10-16

**Authors:** Drew A. Curtis, Christian L. Hart

**Affiliations:** ^1^ Department of Psychology and Sociology Angelo State University San Angelo Texas; ^2^ Department of Psychology and Philosophy Texas Woman's University Denton

## Abstract

**Objective:**

Pathological lying, originally called “pseudologia phantastica,” has an established history within clinical practice and literature, although it has not been recognized as a psychological disorder within major nosological systems. With the movement in psychological sciences toward theory‐driven, empirically supported diagnoses, the current study sought to empirically test whether pathological lying aligned with nosological definitions and could be defined as a diagnostic entity.

**Methods:**

A total of 807 people were recruited (January to October of 2019) from various mental health forums, social media, and a university. Of those recruited, 623 completed the study. Participants responded to a lie frequency prompt, questionnaires about lying behavior, the Lying in Everyday Situations Scale, the Distress Questionnaire‐5, and demographic questions.

**Results:**

Of the participants, 13% indicated that they self‐ identified or that others had identified them as pathological liars (telling numerous lies each day for longer than 6 months). People who identified as pathological liars reported greater distress, impaired functioning, and more danger than people not considered pathological liars. Pathological lying seemed to be compulsive, with lies growing from an initial lie, and done for no apparent reason.

**Conclusions:**

The evidence supports establishment of pathological lying as a distinct diagnostic entity. A definition of pathological lying, etiological considerations, and recommendations for future research and practice are presented.

## HIGHLIGHTS

- Pathological lying exists in a small percentage of people, for whom it causes significant distress, impaired functioning, and danger.
- Pathological lying, distinct from normative lying and prolific lying, has a prevalence of 8%–13%.
- Evidence supports establishing pathological as a diagnostic entity.

The frequency with which people lie varies (1, 2). Considerable research has focused on the normative aspects of lying (1, 3, 4, 5, 6, 7). Deception has been defined by Vrij (3) as “a successful or unsuccessful deliberate attempt, without forewarning, to create in another a belief which the communicator considers to be untrue.” Some studies report that people tell an average of two lies per day (4, 8, 9). Two recent studies, however, discovered that a majority of people reported telling no lies within the past 24 hours, whereas a small subset reported telling numerous lies (1, 2). Although extensive research has explored the normative aspects of lying among the general population, and within psychotherapy (10, 11, 12, 13), the pathological dimensions of deception have been neglected.

Pathological lying (PL) has been referenced in popular culture, although some have suggested that psychiatrists and psychologists know little about the phenomenon (14). PL, originally termed “pseudologia phantastica,” was first recorded in 1891 by psychiatrist Anton Delbrück in discussions of several cases of people who told so many outrageous lies that the behavior was considered pathological (15). Today, there is little consensus for a definition of PL, but many continue to use a definition proposed by Healy and Healy more than a century ago (15). They defined PL as “falsification entirely disproportionate to any discernible end in view, may be extensive and very complicated, manifesting over a period of years or even a lifetime, in the absence of definite insanity, feeblemindedness or epilepsy.”

The *DSM‐5* defines a mental disorder as a syndrome that causes significant distress and impairs functioning (16). Similarly, the *ICD‐10* defines a disorder as a "set of symptoms or behaviour associated in most cases with distress and with interference with personal functions" (17). From these definitions, models of abnormality have been suggested, such as the four Fs: frequency, function, feeling pain, and fatal (18). In comparison with contemporary models of psychopathology, the definition of PL put forth more than a century ago does not fully capture aspects of pathology (15). Therefore, to merge the key elements of the previous definition with psychopathology criteria from classification systems, we suggest that PL should be defined as a persistent, pervasive, and often compulsive pattern of excessive lying behavior that leads to clinically significant impairment of functioning in social, occupational, or other areas; causes marked distress; poses a risk to the self or others; and occurs for longer than 6 months.

PL has not been classified within the *DSM‐5* or the *ICD‐10* (16, 17). The DSM‐5 mentions that deception is a symptom of antisocial personality disorder and is used for external incentive (malingering) and to assume a sick role (factitious disorder) (16). PL is one of 20 items used in the Hare Psychopathy Checklist‐Revised (PCL) (19). However, this item does not serve to provide a diagnosis but to assess lying behavior related to psychopathy.

Research investigating PL is scant. One study of 1,000 young offenders found excessive lying among 15% of males and 26% of females (15). A recent imaging study found that 12 participants who endorsed the PL item from the PCL showed an increase in prefrontal white matter and reduction in gray matter and white matter ratios compared with normal control participants and antisocial control participants (20). Research on PL has mostly involved case studies. Delbrück discussed five case studies, and Healy and Healy identified 12 case studies (15). Across the subsequent 100 years, other PL case studies have been published (21, 22, 23). A comprehensive analysis of 72 case studies (24) showed that PL was equally represented among men and women of average to above average intelligence, typically beginning in adolescence, with some people committing crimes. Although evidenced in case studies, the distinctiveness of PL has been debated (25, 26, 27), with some arguing that PL is a unique disorder (14, 15).

The purpose of the current study was to explore a theoretical model of PL as a distinct psychopathology that meets major nosological definitions, namely a disorder bearing the features of frequency, function, feeling pain, and fatality. On the basis of this hypothesis, we made six predictions: Our first prediction was about prevalence, frequency of behavior, and duration of the condition. We predicted that patients with PL would represent a smaller percentage of the population reporting excessive lying, would report PL as lasting for a longer time than would the general population, and would report onset of the condition as occurring during adolescence. Our second prediction was that patients with PL would report impaired functioning in several areas. Our third prediction was that patients with PL would report more distress from their lying than would the general population. Our fourth prediction was about fatality; we predicted that patients with PL would be more likely to report that their lies put themselves or others in danger. Our fifth prediction was that patients with PL would indicate that their lying was not entirely under their control and that it provided relief from anxiety. Our sixth prediction was that patients with PL would report telling lies for no specific reason and that their lies would tend to grow from an initial lie more so than those of people without PL.

## METHODS

### Participants

We recruited 807 people via Facebook, Reddit/samplesize, Psych Forums, and a university in the southwestern United States to participate in a study on lying behavior. A total of 635 participants completed information beyond the inclusion criteria. Three participants were removed because of reporting an unlikely number of lies told, and nine participants were excluded because they indicated they had lied in response to some of the survey questions. Thus, 623 participants were retained for the analyses.

Participants ranged in age from 18 to 60 years (mean± SD=21.97±7.57) with more female (N=374, 68%) participants than male. The majority of participants were Caucasian and/or European American (N=325, 59%), followed by Hispanic and/or Latinx (N=135, 25%); multiracial (N=41, 8%); African American and/or Black (N=20, 4%); Asian, Asian American, and/or Pacific Islander (N=19, 4%); and Native American and/or Alaskan Native (N=8, 2%). The participants ranged in education, from having no high school diploma (N=6, 1%), a GED (N=4, 1%), a high school diploma (N=210,38%), a college degree (N=309, 56%), a master's degree (N=21, 4%), and a doctoral degree (N=2, <1%). A majority of participants indicated that their annual income was under $25,000 (N=456, 85%), with fewer reporting annual incomes of $25,000 to $49,000 (N=44, 8%), $50,000 to $75,000 (N=18, 3%), and $75,000 or more (N=22, 4%). Although the complete sample of 623 participants drew from a range of ages, ethnicity, education, and income, participants were slightly younger, more Hispanic, more educated, and had lower incomes than the general population.

### Materials

**FIGURE 1.**
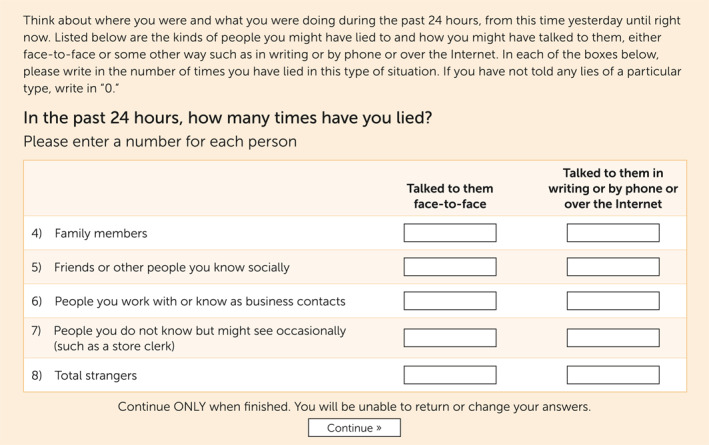
Screen shot of lie frequency assessment^a^ ^a^Source: Serota et al. (1).

Participants were asked about whether they considered themselves to be pathological liars and whether others considered them to be pathological liars. Additionally, participants were given a lie frequency assessment (1, 2) (Figure 1) and other questionnaires. The Survey of Pathological Lying behaviors (SPL) is a nine‐item questionnaire about functioning, feeling pain, and fatal risks of lying behavior that uses a Likert‐type rating scale (1=strongly disagree; 7=strongly agree) (see the online supplement accompanying this article). Internal consistency for the SPL was acceptable (Cronbach's α=0.82). The Survey of Lying Behaviors is a seven‐item survey, reporting on the frequency, functioning, pain, and risks the respondent perceives as related to lying behavior, and has a Cronbach's α of 0.80. The Survey of Others' Pathological Lying is an 11‐item questionnaire, reporting on the frequency, functioning, pain, and risks respondents perceive as related to the lying behaviors of others (Cronbach's α=0.83). The Lying in Everyday Situations (LiES) Scale (28) is a 14‐item scale designed to assess the propensity to lie. The LiES scale has demonstrated high internal consistency, test‐retest reliability, and concurrent validity and displayed high internal consistency reliability in the current study (Cronbach's α=0.88). The Distress Questionnaire‐5 (DQ5) is a five‐item screen, with suggested sensitivity and specificity cutoff points, used to measure general psychological distress among individuals with various psychological disorders (29) (Cronbach's α=0.83). A demographic questionnaire also was provided.

### Procedure

The institutional review board of Angelo State University approved the study. Following written consent, we presented the participants with a prompt and lie frequency assessment (1) (Figure 1). We then asked three questions which we used to identify assignment to the PL or non‐PL condition. Participants in the PL condition completed the SPL, LiES, DQ5, and the demographic questionnaire. Participants in the non‐PL condition completed the Survey of Lying Behaviors, LiES, DQ5, and the demographic questionnaire. Additionally, participants in the non‐PL condition completed the Survey of Others' Pathological Lying if they indicated that they knew someone whom they believed was a pathological liar. All participants were debriefed after participating in the study.

## RESULTS

### Identification of Pathological Lying

We assigned participants to the pathological lying condition if they considered themselves pathological liars or if others considered them pathological liars. Statistics for normally distributed data are likely to be unreliable for handling lie distributions, which have consistently been shown to be positively skewed (1, 2). Thus, we conducted a negative binomial regression to examine the fit of the frequency of lies with self‐identified PL, because it is a robust method for handling overdispersed count data (30). The likelihood ratio chi‐square test indicated that the model was a significant improvement in fit over a null model (p<0.001). Thus, the self‐identification of a PL and non‐PL model was found to be a better fit and was retained.

Of the 623 participants, 83 (13%) indicated that they or others considered themselves pathological liars (35 indicated only self, 27 indicated only others, 12 indicated both, and nine affirmed self or others). A chi‐square analysis revealed a statistically significant difference between people in the PL and non‐PL conditions (χ^2^=335.23, N=623, df=1, p<0.001). Of the 589 participants who responded to the question that asked about having been formally diagnosed by a mental health professional with a psychological disorder, 49 (8%) indicated they were pathological liars. Thus, our prediction was confirmed that PL would occur within a relatively small percentage of the sample (8%–13%).

We conducted a chi‐square test of independence to compare the frequency of formal psychiatric diagnoses among the PL group and those in the non‐PL group and found no significant association (χ^2^=4.42, N=553, df=2, p=0.11). Thus, participants in the PL group were no more likely to have a psychiatric diagnosis than those in the non‐PL group.

We conducted an independent samples t‐test to examine whether there were differences in age between the PL group and non‐PL group and found no statistically significant differences (t=–0.62, df=519, p=0.54). Chi‐square tests of independence found no significant association for sex (χ^2^=0.92, N=551, df=2, p=0.63), education (χ^2^=0.89, N=552, df=5, p=0.97), income (χ^2^=2.77, N=540, df=3, p=0.43), or ethnicity (χ^2^=12.05, N=548, df=6, p=0.06). To examine whether education (inclusion of a college sample) resulted in the differences, we created an education variable to differentiate between those with a college degree or higher and those with a high school education or less. In addition, we used a multivariate analysis of variance (MANOVA) to compare education across lying frequency, distress, functioning, and danger and found no statistical significance for the PL group (F=1.97, df=7 and 58, p=0.07) or the non‐PL group (F=0.49, df=7 and 424, p=0.84).

### Frequency (Prediction 1)

To further test prediction 1, we used a chi‐square test to examine duration of engagement in PL, which demonstrated statistical significance (χ^2^=59.18, N=78, df=24 p<0.001). A majority of participants in the PL group reported engaging in PL for 6 months or longer (N=68, 87%), with more than half (N=42, 54%) indicating they had engaged in frequent lying for more than 5 years (Table 1).

**TABLE 1:** Duration of engagement in pathological lying among participants considered pathological liars (N=78)^a^

Duration	N	%
3 months	10	13
6 months	8	10
1 year	4	5
1–5 years	14	18
>5 years	42	54

a ^a^Data missing for five of the 83 participants considered pathological liars.

We conducted an independent samples t‐test to examine the difference in the number of lies told by participants in the PL group compared with the non‐PL group and found a statistically significant difference (t=7.52, df=588, p<0.001). As predicted, participants in the PL group indicated telling more lies within a 24‐hour period (mean=9.99±11.17, me‐ dian=7, mode=1, N=82, maximum=66 lies, 95% confidence interval [CI]=7.5–12.44, skewness=2.27 [SE=0.27], and kur‐ tosis=7.20, [SE=0.53]) than participants in the non‐PL group (mean=3.09±6.86, median=1, mode=0, N=499, maximum=80 lies, 95% CI=2.49–3.70, skewness=6.79 [SE=0.11], kurtosis=58.43 [SE=0.22]). We conducted a one‐sample t‐test on the PL condition, and a test value of five revealed a statistically significant difference (t=4.04, df=81, p<0.001). Confirming our hypothesis, a majority of participants in the PL group (N=49, 60%) reported telling five or more lies within the past 24 hours. Furthermore, the PL group told more lies in person (mean=6.35±7.75) than over the phone or in writing (mean=3.88±5.82, t=2.85, df=76, p=0.006). We conducted a MANOVA to determine to whom the participants in the PL group reported telling lies and found statistical significance (F=13.71, df=5 and 76, p<0.001). Participants in the PL group reported lying more to friends and social acquaintances than to other people (Table 2).

**TABLE 2:** Lies told by participants considered pathological liars (N=75), by relationship^a^

Relationship	M	SD
Family member (face‐to‐face)	1.40	2.59
Family member (writing or phone or internet)	1.11	2.48
Friends or other people you know socially (face‐to‐face)	2.07	2.71
Friends or other people you know socially (writing, phone, or Internet)	1.53	2.32
People you work with or know as business contacts (face‐to‐face)	1.37	3.12
People you work with or know as business contacts (writing, phone, or Internet)	0.59	1.53
People you do not know but might see occasionally (face‐to‐face)	0.83	1.92
People you do not know but might see occasionally (writing, phone, or Internet)	0.28	0.91
Total strangers (face‐to‐face)	0.76	2.48
Total strangers (writing, phone, or Internet)	0.37	1.08

a ^a^Data missing for eight of the 83 participants considered pathological liars.

We used an independent samples t‐test to compare scores on the LiES between the PL and non‐PL groups and found a statistically significant difference (t=7.09, df=78, p<0.001). Those in the PL group reported a greater propensity for telling lies in their everyday lives (mean=50.80±18.00) than those in the non‐PL group (mean=34.88±12.59).

A frequency analysis revealed that a majority of participants in the PL group indicated onset of PL during adolescence (10‐20 years) (N=48, 62%). A chi‐square analysis showed a statistically significant difference between developmental periods (χ^2^=35.62, N=78, df=2, p<0.001), with most participants reporting that the earliest age they were considered a pathological liar was in adolescence (N=48).

### Impaired Functioning (Prediction 2)

To assess impaired functioning, we conducted a MANOVA with areas of functioning as the dependent variable and the lying condition as the between groups variable. A statistical significance was shown (F=24.09, df=4 and 549, p<0.001). Univariate tests indicated statistical significance between the PL and non‐PL groups' impairment in functioning in occupation (F=15.32, df=1 and 552, p<0.001), social relationships (F=83.88, df=1 and 552, p<0.001), finances (F=27.42, df=1 and 552, p<0.001), and legal contexts (F=29.04, df=1 and 552, p<0.001). Within the PL group, a repeated measures MANOVA showed statistical significance in areas of functioning (F=16.16, df=3 and 72, p<0.001). Pairwise comparisons found the greatest impairment in social relationships (p<0.001) (Table 3).

**TABLE 3:** Pairwise comparisons of impairment in areas of functioning among participants considered pathological liars (N=75)^a^

Function	M	SD	p
Occupation	2.01	1.46	
Social relationships^b^	3.52	2.09	<0.001
Finances^c^	2.32	1.83	0.02
Legal contexts	1.88	1.57	

a ^a^Data missing for five of the 83 participants considered pathological liars.

b ^b^p<0.001 for all other areas of functioning.

c ^c^p<0.05 compared with legal contexts.

### Feeling Pain (Prediction 3)

Participants in the PL group reported greater distress from their lying (mean=3.04±2.02) compared with those in the non‐PL group (mean=2.21±1.65, t=3.44, df=94, p=0.00). Participants in the PL group reported greater general psychological distress (mean=15.76 ±5.11) than the non‐PL group (mean=14.50±4.63, t=2.15, df=539, p=0.03). By using DQ5 suggested cutoff points for sensitivity (≥11) and specificity (≥14), 9% (N=59) and 8% (N=50) of the overall sample were identified as those in the PL group who experience psychological distress, respectively.

### Fatal Danger (Prediction 4)

We used an independent samples t‐test to compare the PL and non‐PL groups on scores of whether their lying put them or others in danger. A statistically significant difference was found (t=5.53, df=82, p<0.001). Participants in the PL group reported that their lying had placed themselves or others in danger (mean=2.76±2.16) more so than participants in the non‐PL group (mean=1.38±1.00).

### Compulsivity (Prediction 5)

To assess compulsivity, we conducted a MANOVA on two items (feeling out of control and for relief from anxiety) and found statistical significance (F=90.47, df=2 and 563, p<0.001). Participants in the PL group indicated that their lying was out of their control more (mean=3.29±2.25) than did individuals in the non‐PL group (mean=1.38± 0.93, p<0.001). Additionally, the PL group felt less anxious after lying (mean=3.51±2.23) compared with the non‐PL group (mean=1.95±1.55, p<0.001).

### Motivation and Growth of Lies (Prediction 6)

We conducted an independent samples t‐test to examine whether the participants in the PL group were more likely than those of the other group to report that they told lies for no reason. A significant difference was found (t=6.13, df=92, p<0.001). Participants in the PL group told lies for no reason (mean=3.73±2.30) more than participants in the non‐PL group (mean=2.09±1.64). Additionally, we used an independent samples t‐test to examine group differences in the belief that their lies grew. The results showed a statistically significant difference (t=6.46, df=91, p<0.001), with those in the PL group indicating their lies grew from an initial lie (mean=3.81±2.22) more so than did those in the non‐PL group (mean=2.15±1.15).

### Pathological Liars Versus Prolific Liars

Prolific liars were identified from the non‐PL sample in a manner similar to Serota and Levine's (2) by using an index of dispersion (D) to decide whether the data fit a distribution. We used a negative binomial regression because of over‐ dispersed data (30). To achieve a dispersion closest to 1, we divided the sample into two groups: those who told zero to two lies (mean=0.66±0.77, D=0.89) and those who told three or more lies (prolific liars) (mean=7.51±10.09, p<0.001). Thus, prolific liars were coded into a new condition variable.

An independent samples t‐test found no significant difference in number of lies told between the PL group and the prolific lying group (p=0.09). However, a significant difference was seen on the LiES (t=6.12, df=224, p<0.001). Participants in the PL group reported greater propensity for telling lies (mean=50.80±18.00) compared with participants in the prolific lying group (mean=38.32±12.03).

We used a MANOVA to analyze areas of functioning between participants assessed as pathological liars and those assessed as prolific liars and found a statistical significance (F=8.86, df=4 and 230, p<0.001). Univariate tests indicated statistical significance for lower functioning among PL participants in occupation (F=4.40, df=1 and 233, p=0.037), social relationships (F=28.44, df=1 and 233, p<0.001), finances (F=7.43, df=1 and 233, p=0.007), and legal contexts (F=12.51, df=1 and 233, p<0.001). An independent samples t‐test found a significant difference between the PL group (mean=3.04±2.02) and those classified as prolific liars (mean=2.49±1.63) with regard to lying causing distress (t=2.08, df=127, p=0.039). Lying was also reported to be more dangerous by the PL group (mean=2.76±2.16) than by the prolific liar group (mean=1.58±1.19, t=4.49, df=100, p<0.001).

### Perceptions of Pathological Liars

Of the participants who did not indicate being a pathological liar, 162 indicated that they knew someone they considered a pathological liar. Participants estimated that these individuals had told an average of approximately 10 lies within the last 24 hours, with five lies as the most frequent response (mean=9.96±15.47 lies told, median=5, mode=5, N=127, maximum=130 lies, 95% CI=7.24–12.68, skewness=5.53 [SE=0.22], kurtosis=37.13 [SE=0.43]). A majority of participants reported either that the person did not have a formal diagnosis (N=67, 42%) or that they did not know whether the person had a formal diagnosis (N=73,46%. Most of these participants reported that the earliest stage of development when the person was perceived as a pathological liar was adolescence (N=83, 52%) or that they did not know (N=42, 26%). A majority of these participants also reported that the person they knew had been telling numerous lies for >6 months (N=121, 76%) or that they did not know (N=35, 22%). A MANOVA revealed a statistically significant difference in areas of functioning (F=590.35, df=4 and 154, p<0.001). Participants indicated that the person's lying had resulted in impaired functioning more in social relationships (mean=5.93±2.14) than in occupation (mean=3.93±2.14), finances (mean=3.84±2.24), or legal contexts (mean=3.27±2.15). An independent samples t‐test was used to compare distress between the PL group (mean=3.04± 2.01) and participants in the non‐PL group who reported knowing someone they considered a pathological liar (mean=4.24±2.00), finding a statistically significant difference (t=−4.32, df=223, p<0.001). An independent samples t‐test also showed a statistically significant difference in danger between those in the PL group (mean=2.76±2.16) and those the participants' considered pathological liars (mean=5.64±1.49, t=−10.59, df=115, p<0.001).

## DISCUSSION

The historical discussion of PL has been robust. Documented case studies have supplied ample evidence of patients with PL behavior in clinical practice. Yet, nosological systems have not classified PL as a distinct entity. Attending to the credence of theory‐driven, empirical approaches to psychopathology (31, 32, 33), the current study provides evidence for PL as a disorder and aligns with the requirements set forth by the American Psychiatric Association for the addition of a new diagnostic category (34). By applying definitions and criteria from psychopathology models, a distinguishable group of people emerges who lie excessively for extended periods and experience impaired functioning, significant distress, and increased danger.

Our findings showed that the participants classified as pathological liars reported telling about 10 lies per day on average, and most reported telling one lie per day. This average is greater than the number of lies told by a normative sample, in the current study, and in previous research (1, 2, 3, 4, 7, 8). Additionally, those in the PL group indicated a greater propensity to tell lies, and the excessive lying had persisted for longer than 6 months, a duration similar to that of other *DSM‐5* disorders (16). Estimates of PL in the current sample ranged from 8%–13%. This range represented those who reported no psychological disorder (8%), those who currently met the DQ5 specificity criteria for distress (8%), and those who self‐identified (13%) as pathological liars.

Our results indicate a distinction between prolific and pathological liars, with the latter endorsing greater distress, impaired functioning, risk of harm, and propensity to lie. The area of greatest impairment in functioning for those in the PL group was in social relationships. This finding was not surprising, because deception often damages trust, especially when used to conceal a transgression (35, 36). Our results provide a strong argument that a definition of PL should hinge not solely on the frequency of the lying behavior, but on the indices of psychopathology.

Participants in the PL group reported experiencing more distress from telling lies. Classic research indicates that lying can reduce distress when behavior is discrepant from beliefs, acting as a distress‐relieving mechanism (37, 38). However, lying can cause distress because it requires a justification for its use (4, 39). For pathological liars, the distress may result from telling lies for no apparent reason, with lies growing from an initial lie, and from concern about discovered deceptions and relational conflict. Additionally, the PL condition indicated greater psychological distress, suggestive of a distinct mental disorder.

The fatal criterion was met because PL was more likely to put oneself or others in danger. An example of lying that may pose a threat to one's safety is if one conceals suicidal ideation during psychotherapy (40).

In addition to the pathological distinctions, we found that those in the PL group indicated that telling lies reduced their anxiety and that their lying was out of their control. Furthermore, PL behavior involves telling lies without a specific reason, with lies growing from an initial lie. Thus, PL contains elements of compulsiveness. The subsequent growth of lies, however, tends to cause more distress.

### Limitations

The current study had some limitations. Participants were recruited from forums that may attract people interested in psychological disorders, which may have increased the PL sample, resulting in a slightly larger prevalence than what may be expected in the general population. However, this method of recruitment was necessary to reach the target sample, because PL is not an official classification within the *DSM‐5*. Although PL is not a formal diagnostic entity, it has been widely discussed by mental health professionals and people who maintain difficulty with lying behaviors. Additionally, our sample included an age range of 18‐60, with an average age of about 22 and many having advanced education. Although we found no statistical difference in a comparison of education levels, use of this sample may have underestimated some of the negative life consequences of PL compared with individuals who are older or less educated. Another potential concern was the self‐report. We used this method because PL has not been established as a diagnostic entity. Additionally, self‐reporters are most likely to seek out treatment. When asking people to report on their lying behaviors, there may be concern that the self‐reports are lies. However, evidence suggests that self‐reports of lying behavior are valid and reliable (1, 2, 41). Halevy and colleagues found that self‐reported frequency of lying behaviors was not significantly correlated to variable response inconsistency or true response inconsistency scales of the multidimensional personality questionnaire‐brief form, with no significant differences found between subjects who were categorized as valid and invalid respondents (41). Finally, although many of the participants indicated they had never been formally diagnosed with a psychological disorder, the current study did not specifically compare PL to various other psychological disorders. Future studies may examine assessment profiles of people with PL behavior to determine convergent and discriminant validity related to other psychopathologies.

### Future Directions

If PL is recognized as a diagnostic entity, researchers would be positioned to examine additional features, etiology, and effective and efficacious treatments. Research may also benefit from exploring clinicians' experiences in treating individuals having PL. Future analyses at the biological level may lead to a deeper understanding of PL. Recognizing PL would equip practitioners to diagnose and treat the condition, thus allowing people to seek treatment. Because PL is not a formally recognized disorder, no systematic studies on the effectiveness of psychotherapy in treating PL have been conducted (27). The utility of implementing cognitive‐behavioral therapy and pharmacotherapeutic options for treating PL is worth consideration (27).

In addition to supporting recognition of PL as a diagnostic entity, the current research adds to deception literature by establishing parameters that distinguish pathological lying from normative lying. The current study showed that categorical distinctions can be made between normative, prolific, and pathological lying. Thus, this study will assist researchers investigating the range of lying patterns.

## CONCLUSIONS

In sum, the current evidence and theory built on existing case studies support establishment of PL as a diagnostic entity. The findings support PL as meeting criteria for a mental disorder, with evidence of a unique, valid, and reliable group of symptoms. We have provided theoretical criteria, etiological markers, and a definition of PL, which should guide clinicians in identifying PL. There are individuals who clearly recognize and report concerns about their own excessive, persistent, and problematic lying behavior. Currently, there is no diagnostic label for these individuals and no specific treatment. Features of PL are distinct and found beyond the forensic population. Definition of a diagnosis of PL would pose low risk of harm and would allow practitioners to formally identify PL and to provide treatment for people looking for relief from its symptoms.

## Supporting information

Supplementary material
