# Supplementary material for: Pathological Lying: Theoretical and Empirical Support for a Diagnostic Entity
Source: Psychiatr Res Clin Pract. 2020 Oct 16;2(2):62–9. doi: 10.1176/appi.prcp.20190046 (PMC9176035; doi:10.1176/appi.prcp.20190046)
Supplement: Supplementary file 1 — Supplementary material [file RCP2-2-62-s001.pdf]

Appendix A
Survey of pathological lying behaviors (SOPL)
1 (*strongly disagree*) to 7 (*strongly agree*) for items 1-7

1. My lying behaviors have resulted in impairment for me in:
   a. My occupation
   b. Social relationships
   c. Finances
   d. Legal contexts

2. My lying causes me significant distress.

3. My lying has put myself or others in danger.

4. My lying is something out of my control.

5. After I lie I feel less anxious.

6. My lies tend to grow larger from an initial lie.

7. Most of the lies I tell are for no reason.

8. What is the earliest stage that you or others considered yourself to be a pathological liar?
   Childhood (3-10 years)
   Adolescence (10-20 years)
   Early adulthood (20-40 years)
   Middle Adulthood (40-60 years)
   Late adulthood (65 years or more)

9. How long have you been telling numerous lies/engaged in pathological lying?
   3 months
   6 months
   1 year
   1-5 years
   More than 5 years
